# Testing the blast response of foam inserts for helmets

**DOI:** 10.1016/j.heliyon.2021.e06990

**Published:** 2021-05-11

**Authors:** S. Bloodworth-Race, R. Critchley, R. Hazael, A. Peare, T. Temple

**Affiliations:** a Cranfield Forensics Institute, Cranfield University, Defence Academy of the United Kingdom, Shrivenham, SN6 8LA, UK; b Centre for Defence Engineering, Cranfield University, Defence Academy of the United Kingdom, Shrivenham, SN6 8LA, UK; c Centre for Defence Chemistry, Cranfield University, Defence Academy of the United Kingdom, Shrivenham, SN6 8LA, UK

**Keywords:** Traumatic brain injury, Energy absorption, Open cell polyurethane foam, Sylgard, Synbone, Shocktube

## Abstract

Modern era combat helmets have different iterations and configurations to offer greater protection from blunt impact or ballistic penetration to suit the theatre of operation, although there are currently no standards for blast protection. Moreover, incorporation of blast protection into the same constrained mass-volume envelope is extremely challenging as there is very little space for a material to absorb or dissipate the shockwave. Foam padding is fitted in contemporary combat helmet designs for comfort and standoff purposes. Examples were subjected to blastwaves generated from an air-driven shocktube, along with open cell polyurethane foam specimens of varying pores per inch and thicknesses to. Whilst the range of samples tested did not afford any superior blast mitigation behaviour over the foam already present in helmets, they exhibited comparable performance with a lower mass. There also appears to be positive correlation between increased mass and increased impulse transmitted through the foam. The literature suggests that multiple mechanisms of damage for blast induced mild Traumatic Brain Injury (bTBI) can be caused by the helmet itself, therefore additional protection from a blunt or ballistic impact may increase the risk of damage from a blast insult.

## 1 Introduction

The most common weapon seen in recent conflicts is the Improvised Explosive Device (IED) with injuries caused by fragmentation and blast as opposed to ballistic penetration or blunt impact [1, 2]. Improvements in ballistic and impact protection of body armour and helmets has led to increased rates of survival, although shifted the focus of the injuries to the exposed head and neck areas [3]. IED blast can cause severe damage to vehicles, structures and personnel, with increasing prevalence of Traumatic Brain Injury (TBI) noticed amongst survivors [4]. Relatively low peak overpressures and short positive impulse time durations can result in Blast-Induced Mild TBI (bTBI), manifesting as anxiety, behavioural changes, even loss of fine motor control, symptoms which can often be confused with Post Traumatic Stress Disorder (PTSD) [5]. In contrast to ballistic injuries they are difficult to attribute to a particular event, often presenting much later, making it difficult to diagnose with confidence [5, 6]. It is estimated that medical care, suicide, and loss of productivity due to TBI cost the US Government $6.2 billion over a two year period following a 2007 deployment [4]. The engineering approach to mitigation of bTBI is concerned with preventing or reducing the peak overpressure transmitted to the brain, therefore the response to this has been improved head protection. For example, modern combat helmets have been through several phases of improvements leading to the inclusion of different variants offering greater protection for blunt impact or ballistic projectiles [7], but incorporating blast protection into the same mass-volume envelope is extremely challenging [8].

Blast damage is caused by the shockwave from an explosive detonation [9]. An idealised blast wave in the form of a Friedlander curve possesses several quantifiable elements; the peak overpressure, positive phase time duration, and the impulse derived from the area under the positive phase, a comprehensive description of blast interaction can be found in the work by Gupta, 2013 [10]. The impulse is the key mechanism for damage to structures [11]. Any degree of confinement can have an amplifying effect as the blast wave cannot dissipate as rapidly as in open air, and is often reflected back [12]. The predominant primary injury mechanism associated with blast is the coupling of the stress wave into the body. The amplitude, time duration and frequency spectrum of the wave all have a direct effect on likelihood and severity of injury, and can be altered by the material response of the armour system worn by the individual [13]. The categories of severity of blast injury are identified in Table 1 along with examples of the blast element associated with causing impact.

**Table 1:** Blast injury categories, the blast element associated with them, and the areas of the human body that are most susceptible [5, 9, 12, 14].

Category	Body part most susceptible	Blast element likely to cause impact
Primary	Gas structures such as lungs, ears, intestines. Does not always present external symptoms.	Interaction between blast wave and the body.
Secondary	Any exposed part of the body. Often the cause of traumatic amputations.	Fragments from the explosive device, structures.
Tertiary	Crush injuries to head and thorax. Limb avulsion injuries.	Gross body displacement, i.e. the blast carrying the body into a wall.

Head mobility or the ‘Bobblehead effect’ also plays a factor in reducing the severity of injury [15]. The repetitive impact of the brain on the inner contours of the skull results from the wild motion of an unrestrained head and is thought to be one of the main pathogenic mechanisms of bTBI. In vivo studies have shown that heads restrained relative to the neck are subject to fewer functional deficits, but immobilising head movement would be an unacceptable and impractical solution in the military context [16]. There are several different potential mechanisms of damage:

• Skull flexure, caused by wave-shaping effects between the helmet and the head [17].
• The brain moving within the Cerebral Spinal Fluid (CSF), impacting on the inner surface of the skull [18].
• Direct transmission and absorption of the blast wave into the brain [18].

Without a clear mechanism of damage to mitigate, and the need to maintain movement of the head, it is very difficult to design a structure to successfully interfere with said mechanism. This study investigated the energy absorption properties of materials commonly used in contemporary combat helmet design, to prevent the blast wave passing through the helmet.

### 1.1 Combat helmet structure

A helmet is designed to protect the wearer from impact (blunt or ballistic) by absorbing and dissipating energy to transmit as little as possible into the head [7]. For combat helmets designed to be worn for extended periods of time with a wide range of head movement being essential, coverage must compromise with practicality of use [7]. In addition to the material of the shell itself, protection is afforded by the maintenance of a standoff distance between the rear face and the skull. This prevents behind armour deformation from impacting the skull, while allowing ventilation and cooling of the head [7]. The volume envelope between the helmet and the head is extremely constrained and cannot be expanded without resulting in a helmet too cumbersome to be practical. Moss, King, and Blackman, 2009 [17] suggested however that a wave-focusing effect may occur in this standoff space between helmet and head, leading to higher overpressures. This in turn could cause increased skull flexure, a potential contributing factor to TBI. The presence of foam pads in the Advanced Combat Helmet (ACH) prevents the underwash of the blast from getting between the head and the helmet, but increases the energy coupled to the head as a result of the greater contact area [19, 20, 21]. Although there are multiple potential bTBI mechanisms of damage [17, 18] that could be attributed to the helmets themselves, this is not explored further in this work. Instead, focus is on the materials themselves rather than geometry or method of application. The existing properties can offer protection from secondary and tertiary blast injuries such as fragmentation and impact, but due to the open face none are thought to be capable of preventing bTBI [18].

Strain rates for blast loading conditions are estimated to range between 2000-10000/s, presenting a very difficult engineering challenge to mitigate blast across all strain-rate domains, as materials very often have strain rate dependent properties [22]. In some earlier helmet models, standoff is achieved by a suspended net inside the crown, in others a series of polyurethane foam pads are fitted to provide additional comfort. The foam acts as a shock absorber for blunt impact, although the effectiveness of the material as a blast mitigation layer is unknown [11]. Padding is also advantageous when using the chinstrap to secure the helmet to the head. Whilst securing the helmet to the head with a chinstrap aids protection from ballistic and blunt impacts, the same structure can cause additional damage via different mechanisms. The chinstrap secures the helmet in place but when the helmet is lifted by a blast wave the chinstrap causes it to bounce back down onto the head. The forces transmitted from the strap can follow paths towards the cerebellum. The latter direction of travel is thought to cause TBIs [7].

Foams are innately variable in structure and performance, as the manufacturing process does not allow control of all variables [23]. They are typically manufactured by injecting gases or foaming agents under pressure into the base polymer, expanding to generate pores, and then thermosetting the material [23]. Variability within a single sheet of foam is high, and in plane vs out of plane properties will vary with orientation depending on the foam symmetry, for example foams are commonly twice as stiff in the rise direction as the normal [23, 24]. The key differences between open and closed cell foams can be found in [24, 25, 26].

In open cell polyurethane foams of the type seen in combat helmets, the collapse mechanism is elastic buckling. Cell collapse ends once opposing cell walls begin to touch, and structure densifies with stiffness increasing rapidly [27]. This is the point of densification, where further force compresses the material itself [27, 28]. Foam deformation in response to loading is not uniform, as it first occurs in the weakest point of the material, which cannot be predicted due to the random nature of bubble formation during manufacture [29]. To provide effective protection within a helmet, all energy absorption must occur prior to densification, after which all remaining kinetic energy would be transferred to the head [30]. Elastomeric collapse by elastic buckling is a recoverable mechanism, and repeated compression causes fatigue damage, particularly in polyurethane [28].

### 1.2 Energy absorption

Foams absorb more energy than their base solid material for a given max stress, as the plateau region allows large energy absorption at near constant load [31]. Elastomeric materials show damping or hysteresis so not all external work absorbed is recovered. If the density is too low, the foam bottoms out with a sharp increase in force before all energy is absorbed. If too dense, then force will exceed the critical value before enough energy has been absorbed. The ideal foam has a flat plateau stress just below the critical damaging level, with area under stress-strain curve up to densification strain equal to kinetic energy absorbed [31].

Energy transferred from blast pressure wave to a weaker foam is higher than energy transferred to foam with higher crushing strength. Foams with lower crushing strength absorb higher energy from the incident blast pressure wave, although require a longer length of compaction to fully dissipate energy [32]. This equates to requiring a thicker layer of foam for a more flexible material of the type used in helmets. Sandhu et al [33] showed that for increasing thicknesses an open cell rubber foam, the peak overpressure transmitted is initially amplified until a point of critical thickness is reached, at which point the transmitted pressure decreases and blast mitigation is observed. For the material used the critical thickness was 40 cm, a size which would not be practical to implement in helmets where the typical thickness of a comfort pad was observed to be 20–30 mm. Polyurethane foams are widely used for comfort padding including in combat helmets, as they are lightweight, readily available and low cost. However, Sandhu et al discovered that they also amplify the peak overpressure as the thickness increases when tested using a shocktube, but blast mitigation has been observed in explosive experiments [33]. This discrepancy highlights the importance of creating as representative an environment as possible during experimentation, to allow reasonable extrapolation of data and meaningful conclusions. The behaviour of foams is strain-rate dependent with stiffer behaviour corresponding to the increase in strain-rate, therefore energy absorbed for any given strain-rate is not all encompassing [34, 35, 36, 37, 38, 39, 40].

Whilst there has been substantial research into blunt impact and ballistic penetration resistance of helmets, much less work has been published on the blastwave in isolation, and lesser still utilising experimental work to validate simulations [17, 21, 41, 42]. As there appears to be little to no deliberate incorporation of shockwave absorption within a typical combat helmet structure, this work examines whether any such properties are coincidentally present within helmets that utilise foam padding. The study focussed on the material response of potential alternatives to helmet foams, assessing whether any improvements to the performance of currently available comfort pads could be made. Helmet geometry, head orientation, and multi-hit effects are all considered to be outside the scope of this work, as are the types of helmets and body armour worn by EOD operators as they have very different structures and mobility requirements to combat helmets.

## 2 Experimental set up

### 2.1 Shocktube

**Figure 1:**
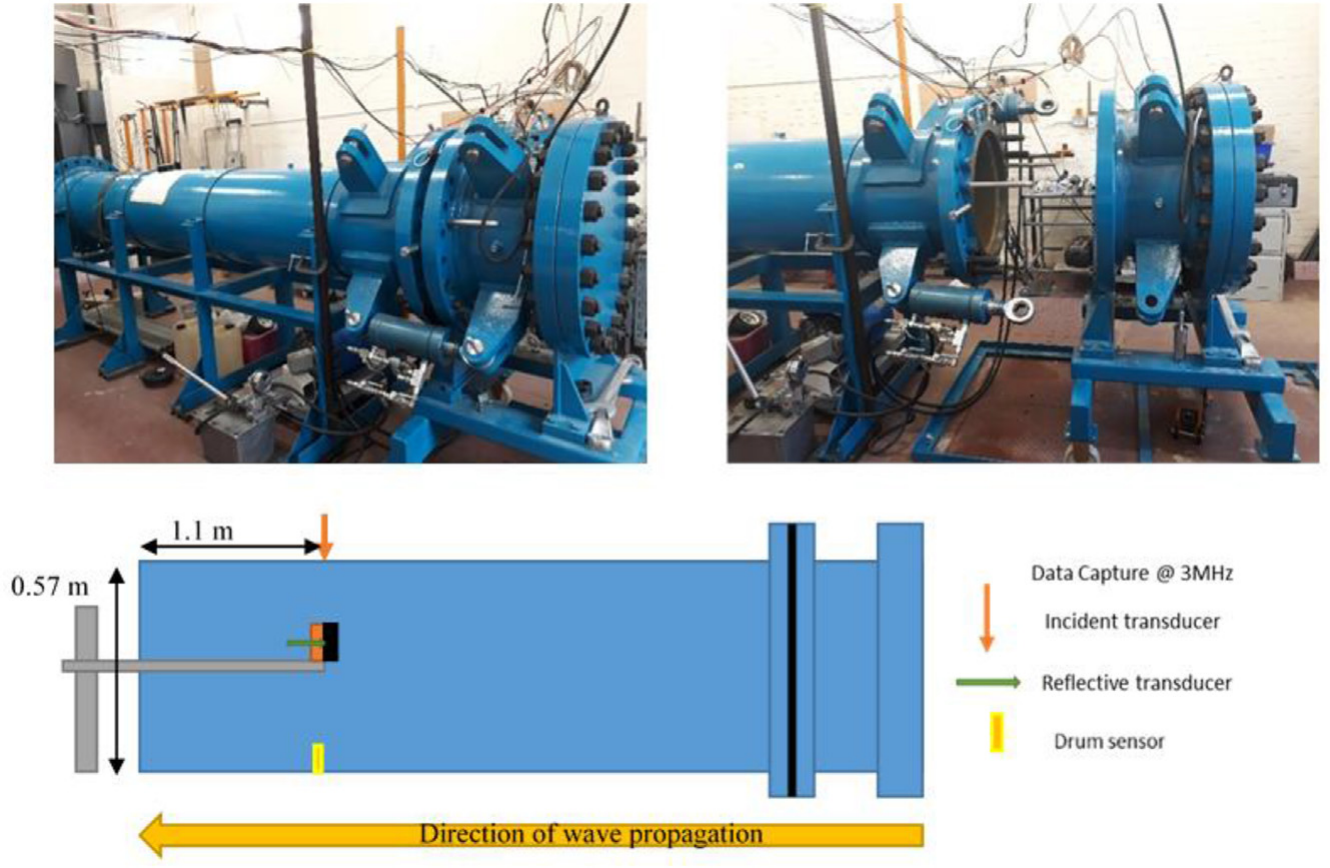
(L) Shocktube (R) Driver chamber opened for insertion of diaphragm (Bottom) Experimental schematic showing shocktube dimensions, instrumentation type and placement, and direction of shockwave travel – Not to Scale.

The experiments were conducted using a shocktube seen in Figure 1 with external and internal diameters of 600mm and 565mm respectively. Total length was 4550 mm, consisting of a 4000mm driver chamber, and a 500mm driver chamber divided by a 50mm intermediate chamber. Diaphragms were cut from 125 μm mylar sheet using metal stencil and scissors, then secured to rubber gaskets with double-sided tape. Individual diaphragms were then placed in the intermediate chamber and sealed with the application of 100 bar hydraulic pressure. The driver chamber was pressurised using compressed air until diaphragm rupture, at a mean pressure of 90 ± 3 kPa. Due to the fixed volume of the driver chamber (0.1339 m^3^) the time duration of the incident blast wave was nominally 6 ms. Data from two Kistler 603B 0-200 bar piezoelectric pressure gauges was captured at a frequency of 3 MHz with Prosig P8020 coupled with a charge amplifier, recording for 30 ms per test. Both the incident (initial) and reflective (transmitted through the material) blast waves were measured as shown in the experimental schematic of Figure 1. Data capture was triggered using a physical disk gauge (drum sensor) located on the base of the shocktube in line with the front face of the transducer as indicated in Figure 1, using a pre-trigger time of 30 ms to ensure the event was recorded.

**Figure 2:**
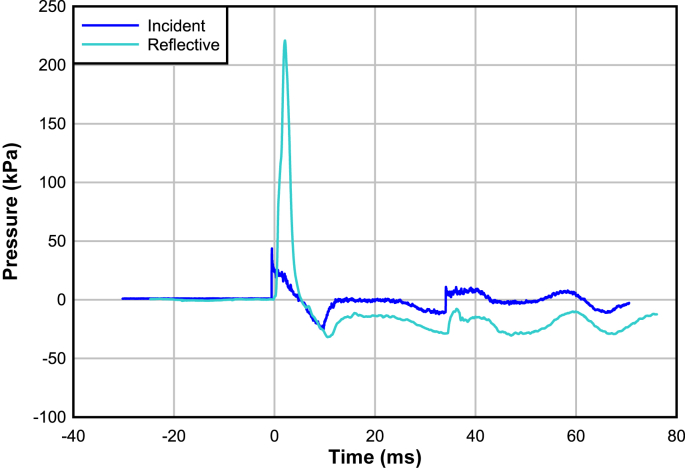
Example P-t output corresponding to a Friedlander curve, showing the sharp amplification of the incident overpressure, slight increase in time duration of the positive phase, and the corresponding shape of the negative phase.

Data was post-processed using DPlot plotting software version 2.3.3.8 to plot pressure-time (p-t) graphs, with the implementation of a 5-point smoothing filter to reduce noise. Where required the baseline shift feature was used to zero all curves about the x-axis to ensure accurate peak overpressure readings and enable direct comparison between datasets. The integration feature was used to ascertain the positive impulse values from the p-t curves. As in blast injury mechanics [43], reflective peak overpressure was used as the primary metric of performance. The shape of the negative phase of the waves was not analysed quantitatively, but any deviation in shape may indicate changes in behaviour such as disruption to the jig or transducer movement (see Figure 2).

### 2.2 Sample jigs

Testing was conducted across two test series, the initial on a rigid jig to assess material behaviour and provide an indication of the factors affecting blast mitigation, e.g. density or porosity. The structure of the Test Series 1 (TS1) jig was a wall of plastic construction bricks with 3D printed central blocks to allow the insertion of the transducer, with the bricks possessing the advantageous properties of modularity and high manufacturing tolerances. Reinforced tape was applied to secure the structure to the supporting bar, providing the necessary compression to prevent blocks detaching under the blastwave. The dimensions were matched to the size of the foam samples, with sufficient thickness to withstand the blastwave without disintegrating (32 mm). Foam specimens were secured on either face to the jig and the para-aramid to the foam by using fibre-reinforced double-sided tape, placed at the edges to avoid obstructing the transducer.

**Figure 3:**
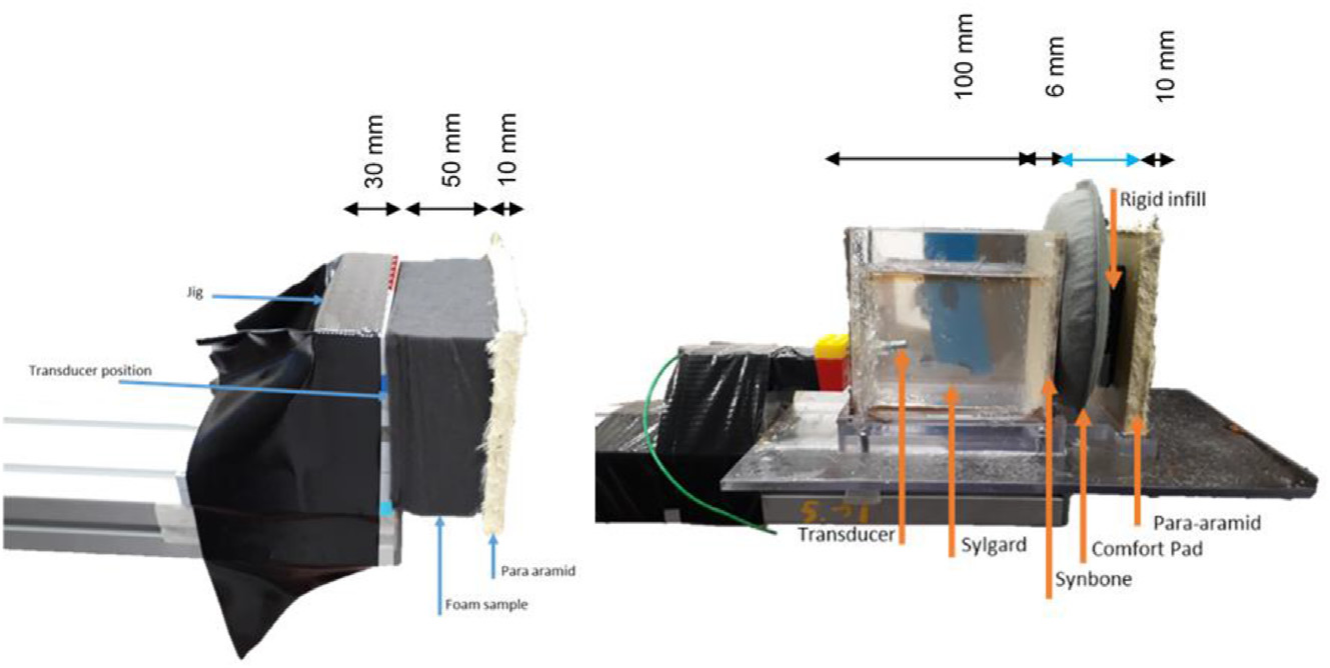
(L) Test Series 1 jig, to capture the differences in transmission through foam samples (R) Test Series 2 with materials simulating layers of a combat helmet, skull, and brain to capture the differences in energy transmitted through the foam and skull, into the brain.

The second test series was conducted on a first order approximation of a human head, to serve as a more representative environment. This was done using the Synbone cancellous bone simulant to replicate the skull, and Sylgard brain tissue simulant, the two-part elastomer mixed 1:1 by volume. The dimensions of the Test Series 2 (TS2) jig were selected to approximate the size of the brain in a frontal orientation to the blast. To allow variable thickness of foam the para-aramid was supported by a slot machined in a movable section of the baseplate. Tape was applied along the sides of the jig to prevent the para-aramid shifting position during the blast. Figure 3 shows the structure of the jigs.

Key points to note are the unconfined rear face of the Sylgard on TS2, to mimic transducer placement in the centre of the brain, and the rigid face of the jig in TS1. TS1 is analogous to capturing the pressure transmitted through the helmet to the skull, whilst TS2 would provide an approximation of the pressure transmitted into the centre of the brain. The lack of a back plate prevents the interference of the reflective wave rebounding off the rear face of the ‘skull’ (the portion of the tensile wave reflected at the interface of the Sylgard-air boundary is considered negligible).

### 2.3 Sample preparation

**Figure 4:**
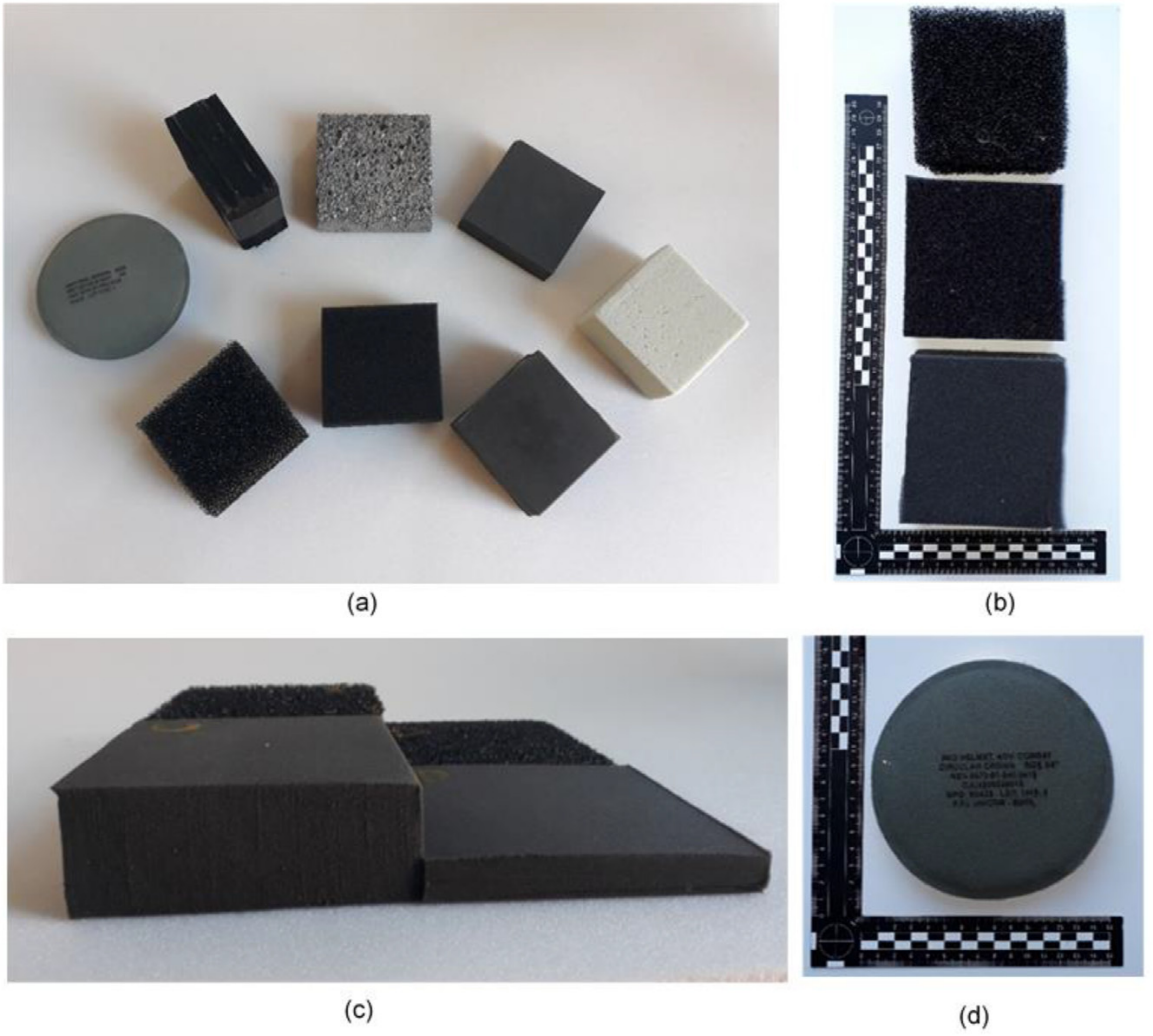
(a) Full range of TS1 samples (b) top down: 10, 45, 90 PPI Reticel samples (c) Compressed 10 and 90 PPI Reticel samples (d) Example of a comfort pad used in these experiments.

Table 2 lists the different types of foam used across both test series, where the majority of samples were open cell polyurethane foams of different pores per inch (PPI) supplied by Reticel, UK. These samples were used as they were inexpensive, readily available, and their properties were well investigated in wider literature [23, 33, 44]. Due to the similar mass and density ranges of the Reticel foams, additional foam specimens were investigated to ascertain if any notable relationship between mass and density also existed (see Figure 4a). The sheet thickness was consistent at 50 mm, with nominal sample dimensions were set at 100 × 100 × 50 mm as seen in Figure 4b, to simulate a sheet or layer inside the helmet. This also ensured sufficient coverage of the jig surface area and transducer to prevent the incident wave from washing over the edges of the foam and interfering with the reading of the reflective wave.

**Table 2:** Physical and mechanical properties of all materials used in TS1.

Descriptor/Variable	10	45	90	Metal	Green	Grey	Black	Comfort Pad
Material	Polyurethane - Reticel			Aluminium	Polyurethane			
ρ (kg/m^3^)	29.03	27.65	28.01	417.9	48.53	30.66	159.5	226.1
PPI	10	45	90	Not examined				
Thickness (mm)	50	50	50	33	50	30	50	20
Structure	Single layer of bare foam						8 × 4 mm layers	Fabric cover
Mass (g)	14.6	14.4	14.2	173	24.3	92	77	78

To rule out the influence of the foam base material, 10 and 90 PPI foams were subjected to a thermo-mechanical process to induce a density change. A jig was constructed from aluminium sheet and the samples were compressed and held with locking pins, then heated for 60 min at 180 °C in a fan assisted oven. They were then allowed to cool to room temperature and removed from the tray, maintaining the new shape. The resulting sets of samples were compressed by 75% and 25% of the original 50 mm thickness (see Figure 4c), properties shown in Table 3. Notation is given by ‘C’/degree of compression/PPI.

**Table 3:** Physical properties of compressed foam samples used in TS2.

Variable	C2510	C7510	C2590	C7590
PPI	10	10	90	90
ρ (kg/m^3^)	41.42	111.4	39.15	103.9
Nominal Thickness (mm)	37	13	37	13

### 2.4 Material characterisation

The 10, 45, and 90 PPI open cell foams were compressed by an Instron mechanical test machine using a 1kN load cell. Compression was conducted in the parallel and normal directions to the axis of cell elongation, to identify the Young's modulus in the direction of the samples that underwent blast loading. For each foam, 10 cubes of 50 mm per side were tested and the compression-load results converted into Stress-Strain graphs using Eqs. (1) and (2). The compressed 10 PPI and 90 PPI foam samples in Table 2 were also tested and plotted in the same manner.

(1) $$\sigma =\frac{F}{A}$$

Where:

σ = stress (Pa)
F = force, (N)
A = area (m^2^)

(2) $$\in =\frac{x}{l}$$

Where:

ε = strain
x = extension (mm)
l = original specimen length (mm)

## 3 Results

### 3.1 Static compression testing

**Figure 5:**
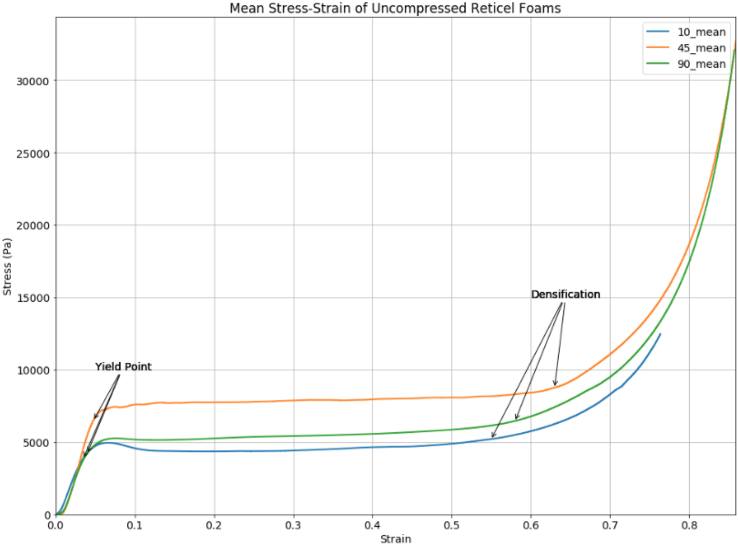
Mean Stress-Strain of 10, 45, and 90 PPI uncompressed Reticel foams, with clearly identifiable yield points used to calculate the Young's modulus from the gradient of the linear region.

For each non-compressed PPI category of foam two distinct stress-strain profiles were observed, corresponding to the orientation of compression axis relative to pore alignment [31]. The profile of the relevant orientation to the blasted samples is shown in Figure 5 with the phases marked. The Young's modulus were calculated from the gradient of the linear region up to the identified points, shown in Table 4.

**Table 4:** Young's Modulus of Reticel foam samples, calculated from the linear regions of the stress-strain curves, and the energy absorbed per unit volume.

Sample series	E (kPa)	Energy absorbed per unit volume
10 PPI	122.77	2422.84
45 PPI	175.63	4725.48
90 PPI	127.27	3032.15
10–37mm	19.38	1384.03
90–37mm	37.78	1727.88

The mean stress-strain curves for each set of Reticel foams were plotted, the elastic, plateau, and densification regions can be clearly distinguished in Figure 5 and the Young's modulus in Table 4 measured from the gradient of the elastic phase ending at the respective yield points.

**Figure 6:**
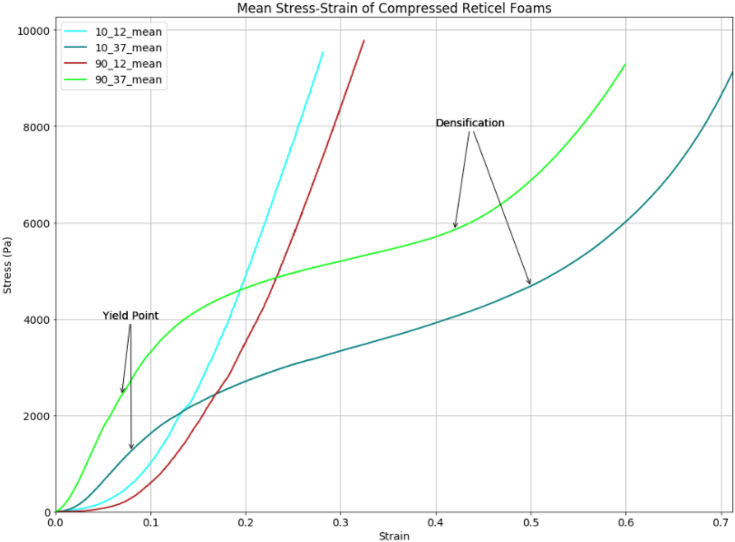
Mean Stress-Strain of 10 and 90 PPI compressed Reticel foams, yield points are much harder to identify clearly for the 37mm thick samples, and completely different behaviour is seen from the 12mm thick samples.

The thermomechanically compressed 10 PPI and 90 PPI samples which underwent shocktube testing were also tested to assess the effect of artificial increase in density on material properties. These results are shown in Figure 6 with the Young's Modulus and energy absorbed in Table 4. The 12mm thick samples did not demonstrate a linear elastic or plateau phase therefore these parameters could not be calculated. All stress-stress testing results were plotted using Python.

### 3.2 Shocktube test series 1

**Figure 7:**
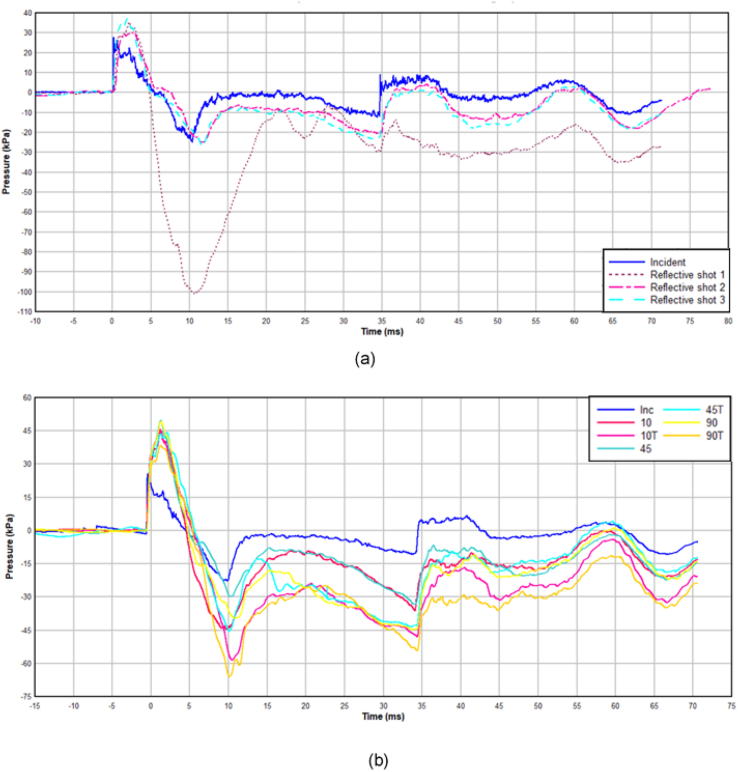
TS1 (a) Comfort pad P-t curves. (b) Reticel mean P-t curves, those datasets incorporating the para-aramid in front of the foam denoted with ‘T’.

Figure 7a shows the repeated response of the comfort pad in comparison to the incident wave. The rupture pressure of the diaphragms was nominally 90 kPa, with the blast wave dissipating as it propagates down the length of the shocktube. The incident pressure measured at the same distance along the tube as the samples was nominally 28 kPa for all experiments. As there was only one comfort pad available, all tests were conducted on the same sample. No significant change in performance was observed throughout the testing that might have indicated a fatigue effect.

The responses of the first round of testing on the Reticel foams are also shown in Figure 7b which illustrates the effect of placing the para-aramid (a 60/40 resin/fibre composition) sheet in front of the foam to simulate the helmet. Those shots conducted with the para-aramid attached to the front face of the foam are denoted with the suffix ‘T’. Visual examination of the foam samples post-impact showed that there was no apparent deformation or damage from a single blast visible to the naked eye.

Due to the variation in diaphragm rupture pressure direct comparison of the raw peak overpressure and impulse between different experiments would not be appropriate. Each shot was normalised with respect to its incident wave impulse, using Eq. (3). The resulting normalised impulses and pressures are given in Table 5, the comfort pad measured the lowest normalised reflective impulse and pressure. The T series' had very similar performance to their corresponding bare foam series' all within one standard deviation. All data points are within one standard deviation of the 45 PPI value, the sample with the largest variation.

(3) $$\text{Normalised}\;\text{Impulse}=\frac{\text{Reflective}}{\text{Incident}}\times 100$$

**Table 5:** TS1 Normalised reflective impulse and pressure.

Sample	Mean Normalised Reflective Impulse (kPas^−1^)	SD (kPas^−1^)	Mean Normalised Reflective Pressure (kPa)	SD (kPa)
Comfort pad	151.46	1.11	12.74	-
10	190.31	47.03	46.03	8.39
10T	193.46	35.39	42.37	6.81
45	185.98	22.61	47.81	3.56
45T	242.45	82.24	44.84	3.79
90	187.79	10.38	49.54	1.81
90T	178.84	16.52	34.51	3.92

**Figure 8:**
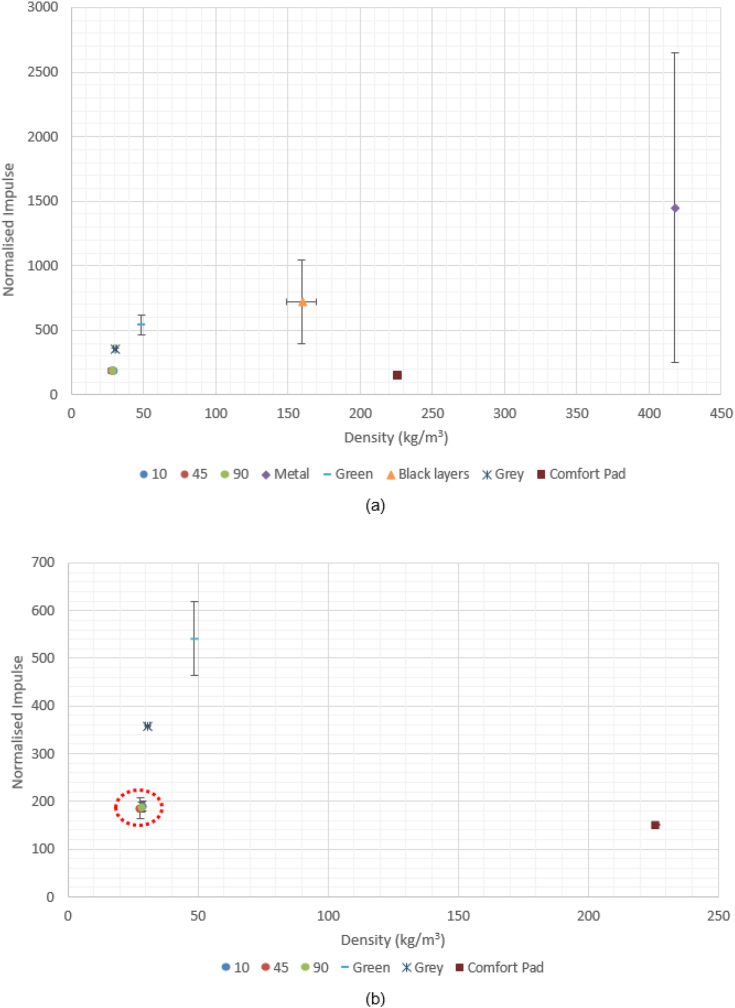
(a) TS1 Normalised Impulse vs Density, with Reticel foams highlighted (b) Enlarged view of Reticel foam performance, comparable to the comfort pad.

Normalised impulse data is plotted against density as shown in Figure 8a, with the Reticel foams highlighted. Figure 8b shows the focused view of the foams and comfort pad for comparison. A clear positive linear correlation can be seen between increasing density and normalised impulse, although the comfort pad appears to be the outlier to this trend. There was no substantial variation in density between the Reticel samples with different PPI values, as shown in Table 6. Density was calculated from the cubic samples used for static compression characterisation, measured prior to compression.

**Table 6:** Density of the 10, 45, and 90 PPI uncompressed Reticel foams.

	Mean (kgm^−3^)	SD (kgm^−3^)
10 PPI	30.24	0.92
45 PPI	28.02	0.45
90 PPI	28.67	0.61

The relationship between impulse and peak overpressure is explained in detail in [45]. In summary, the magnitude of the peak overpressure is not the sole indicator of the energy imparted by a shockwave. The longer the time duration of the impulse, the greater the energy imparted and the greater the damage inflicted. Increasing either the peak overpressure or the duration with increase the impulse, allowing the capture of both variables in a single mechanism. Impulse is used throughout this work, but as the time duration remains the same, any variation in impulse results from a change in the peak overpressure. Therefore this study will use impulse as the key parameter for characterising blast response.

**Figure 9:**
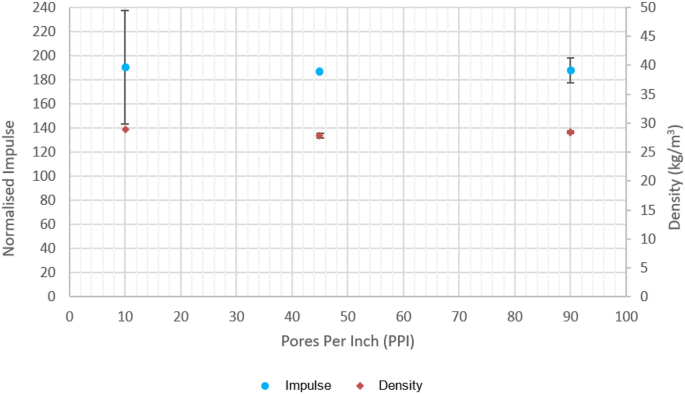
TS1 Effect of porosity on normalised impulse and foam density.

Figure 9 shows the impact of increasing porosity on the normalised impulse and density of the 10, 45, 90 PPI foams. Data points are the mean values for five tests for each porosity. For this range of polyurethane open cell foams it can be seen that there is no correlation between PPI and normalised impulse or density, all data points are within one standard deviation (shown by the error bars where visible) of each other within their respective series. The larger error bars on the 10 PPI sample set can be attributed to the nonhomogeneous nature of foam, as discussed in the literature [46]. As the individual 10 PPI cells are much larger than those in the 45 and 90 PPI foams, it is suggested that a percentage size variation of a single cell will have a greater impact on bulk material properties.

**Figure 10:**
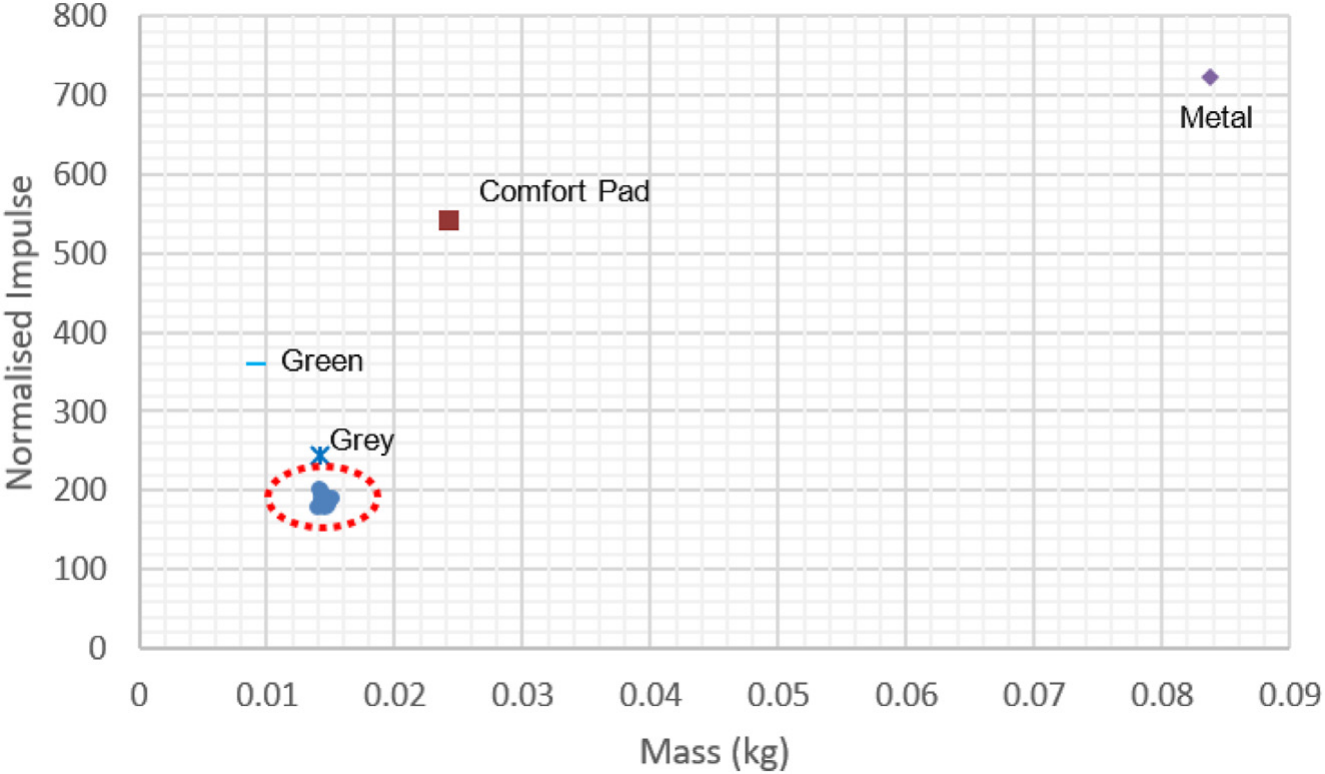
TS1 Positive correlation between foam mass and normalised impulse, the Reticel cluster highlighted.

The effect of increased mass on normalised impulse is shown in Figure 10. There is a positive linear relationship between increased mass and normalised impulse. The two outliers identified are the green closed cell foam and the comfort pad, whilst the Reticel foams can be seen clustered with very similar performance at the bottom left of the plot.

### 3.3 Shocktube test series 2

**Figure 11:**
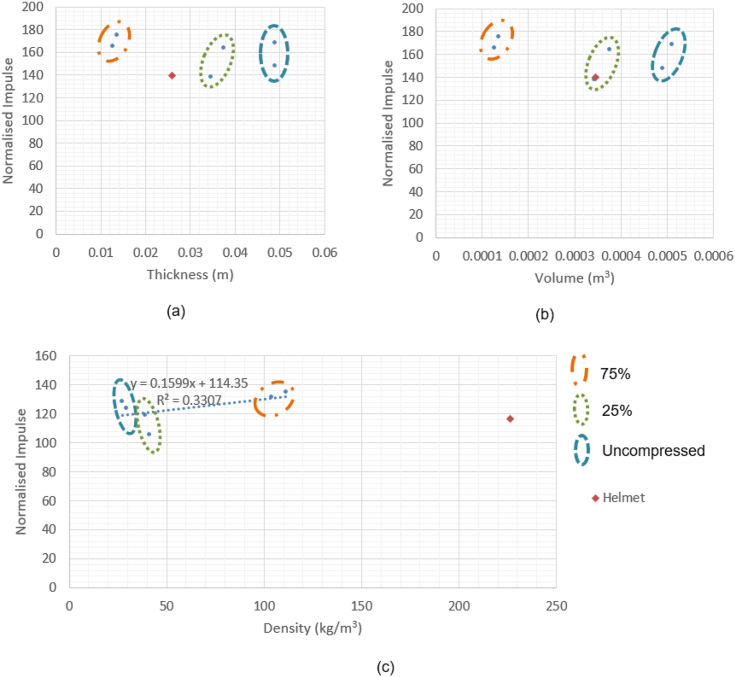
TS2 Effect of increasing (a) Thickness (b) Volume (c) Density of Reticel foams on normalised impulse.

TS2 encompassed the testing of the compressed foams shown in Figure 4d, fresh 10 and 90 PPI foams samples at their original thickness, and the comfort pad for comparison, all on the Sylgard jig. Figure 11 shows the effect of varying parameters (thickness, volume, and density) of the samples on normalised impulse, all points plotted are the mean value of three samples.

Each ‘density pair’ of 10 and 90 PPI foams is ringed i.e. the 10PPI and 90PPI samples that were compressed to 25% of their original thickness are grouped in orange, and as can be seen they have very similar performance to each other, in the same manner as TS1. It can also be seen that increasing thickness or volume of the foam does not trigger any change in performance. There is however a smaller density range used in TS2 due to the exclusion of the aluminium and closed cell foams. Due to their differences in material and construction, they were judged unsuitable for direct comparison with the Reticel foams. These foams were included in the first test series only as indicators of a mass/density effect, as they would be impractical choices for inclusion in a helmet. In all three figures it can be seen that the comfort pad has comparable performance to the open cell foam samples in all their configurations.

## 4 Discussion

It is expected that there will be an increase in normalised impulse with increase in density of a foam sample, as explained in the literature [33]. For a positive blast mitigation effect in the context of this study, a value of normalised reflective impulse as a percentage of incident impulse (calculated using Equation 3) measurably below 100 would be required. Values near to 100 would indicate transmission of the reflective wave straight through the foam without any change in peak overpressure or positive phase duration. Values above 100 indicate that the specimen amplifies the reflected blastwave.

### 4.1 TS1

The duration of the positive impulse was observed to be the same across all measured waves, both incident and reflective (6 ms). As shown in Figure 7b, all open cell foam and para-aramid combinations amplified the peak overpressure by an approximate factor of 2. Variation in the magnitude was observed in the negative phases, which is likely attributed a combination of the following factors:

• Inherent variability of foams, each shot was conducted on a fresh sample where feasible.
• Ringing effect from the metal support stanchion.
• Human variability in positioning of jig within shock tube, and of securing the sample to the jig face each time.
• Transducer movement within the jig, due to clearance fit.
• Variation in the rupture pressure of the diaphragms altering the magnitude of the negative phase.

Figure 8 showed that normalised impulse increased linearly with density of foam. A possible cause of this is the increase in mass associated with the increase in density for a given volume, which would result in a greater mass of foam being accelerated into the ‘head’. Figure 8 also shows the effect of increasing density amongst the comparable polyurethane foams only, with the performance of the comfort pad included for reference, as it was classed as a complex system with the fabric cover. Similar values for normalised impulse can be observed between the open cell foams and the comfort pad, with the former group possessing a lower density.

The Reticel foams were all found to have the same density and performance regardless of porosity as shown in Figure 9, with an amplification effect almost doubling the peak overpressure upon transmission through the foam. This led to the testing of the other polyurethane and metal foams to encompass a wider density range within the sample population. The black layered and the aluminium foam specimens were not directly comparable to the other specimens due to the differences in structure and material, but provided an indication of the positive correlation seen in Figure 8.

The key findings from TS1 were that the density and/or mass effect was responsible for the increase in normalised impulse, therefore variation of density was investigated further on the Sylgard jig, which provided a first order approximation of the human head.

### 4.2 TS2

The only materials tested in TS2 were the comfort pad and the 10 and 90 PPI foams at varying degrees of compression. The green, grey, black and metal foams were excluded as they would not be suitable for fulfilling the comfort requirements in a helmet. The green foam and aluminium foam were not capable of elastic recovery, and the other samples were considered too dense to be comfortable or absorb sufficient energy from a blunt impact.

As seen in Figure 10, there was no measurable influence of the changes in thickness, volume, or density on the normalised impulse measured by the transducer when embedded in the Sylgard. It does however clearly show that the 10 and 90 PPI foams have very similar performance and maintain similar densities to each other when compressed, with each pair highlighted. The 10 and 90 PPI foams were used to assess whether the behaviour of the open cell foams was consistent on the Sylgard, confirming that the porosity did not influence the performance. That is to say, the reflective wave impulse was still approximately double that of the incident impulse for all samples. The comfort pad demonstrated the lowest normalised reflective impulse of all samples. Figure 10 supports the findings of Figure 8 in showing that the increase in normalised impulse corresponds to the increase in mass. This would correlate to the increased mass being accelerated into the head, coupling a greater amount of energy into the skull compared to a bare head, due to conservation of momentum.

In contrast to literature [47] and the results from TS1, there was no significant positive correlation between increasing density and normalised impulse or pressure. A possible contribution to this marked change in behaviour is suggested to be the difference in construction of the jigs, and the transducer placement. As the Sylgard structure possessed an unconfined rear face, and the transducer was embedded within the body of the Sylgard well behind the Synbone it is possible that further energy was absorbed by the Synbone. Due to the size limitations of the internal shocktube diameter it was not possible to capture any high-speed video footage to observe any such potential dissipation of the blastwave within the Sylgard itself. There was also a much smaller range of densities in the sample population of TS2 so to confirm if the correlation is simply too small to capture in a smaller population, denser polyurethane open cell foams with the same dimensions should be subject to the same test procedure.

It is also possible that the Sylgard would absorb energy from the shockwave as it passes through the material, and that placing the transducer in direct contact with the rear face of the Synbone would exhibit different behaviour. In order to establish any energy absorption properties of Sylgard, multiple transducers should be used placed at intervals along the direction of shockwave propagation. Whilst it is acknowledged that impedance will have an effect on the way the shockwave propagates through the different materials and reflects at material boundaries, it is currently outside the scope of this research and should be investigated in further work.

Figure 11a and b show that the open cell foams possessed very similar characteristics of thickness, volume, and performance to the comfort pad. Figure 11c however shows that whilst there is no significant corresponding increase in normalised impulse with density, the open cell foams achieved comparable performance to that of the comfort pad, at up to 1/8th the density. As the samples all had the nominal mass prior to compression, this supports the finding that it is increase in mass which causes increase in normalised impulse. This in turn accounts for the correlation between increasing density and impulse, as increase in volume had no impact on performance.

This corresponds with explanations posed in literature [33], which suggest that the implementation of foam padding amplifies the peak overpressure transmitted to the head, as the larger contact area between head and helmet more effectively couples the energy to the brain than would occur in a helmet without padding. As the samples used in this study exhibited amplification effects of the reflected shockwave, they do not possess sufficient energy absorption capacity prior to the point of densification. The addition of any extra mass into the system will increase the momentum impacting against the head. Practically speaking, a helmet which utilises a suspension net or webbing system to maintain ballistic standoff will reduce the energy transmitted directly through to the head. The underwash of the blast may however then induce wave-shaping effects between the helmet and the skull, causing skull flexure and resulting bTBI, whereas the presence of padding can prevent underwash [17].

While data can be plotted in a Pressure-Impulse relationship to assess injury, without the formation of a threshold for mTBI for comparison, such an approach would not be meaningful. Rutter 2019 [48] postulates that the threshold pressure for mild bTBI lies at 17.7 kPa with impulse of 7.2 kPa ms^−1^, overlapping the threshold for eardrum rupture.

The apparent lack of correlation between thickness and impulse does not fit within the domain of the literature, which poses the theory of critical thickness for blast mitigation [33]. This deviation could be attributed to the relatively small range of thicknesses used in these experiments, compared to the 40 cm critical thickness postulated in the relevant study. This study found that increasing thickness (and therefore mass) of an open cell rubber foam caused an amplification of the reflective impulse, until the critical thickness threshold. Beyond this, a continuing reduction in amplification was observed and ultimately the reflective impulse was reduced below that of the incident, achieving successful blast mitigation. Were further work conducted looking at increased thicknesses of the Reticel foams, this pattern may well emerge, and a critical thickness could be found, but this would be well in excess of the practical mass-volume envelope for a combat helmet.

### 4.3 Static compression testing

The 10, 45, and 90 PPI profiles shown in Figure 5 have clear linear-elastic regions, yield points, plateau regions, and points of densification, but these features are less identifiable on the graphs of the thermomechanically compressed samples. The 37mm samples of both 10 and 90 PPI have a diagonal plateau region but still exhibit Young's slopes and point of densification. Although the yield points are less obvious due to the diagonal shape of the plateau region. This can be attributed to the gradiated structure of the samples following thermomechanical compression, leaving changes in relative density throughout the material. Physical damage was observed on the rear surface of the 37mm thick 10 PPI samples following stress-strain compression testing, suggesting that this material would not be suitable for a multi-hit purpose. The dynamic response of a material is independent of the stress-strain data in a blast event however, therefore micro changes on the surface material caused by compression do not effect the macroscopic behaviour under blast loading.

12 mm samples both 10 and 90 PPI have no linear elastic phase or plateau region and hit densification almost immediately on application of load, showing that they were permanently deformed by the thermomechanical compression, and no longer capable of absorbing energy. This suggests that artificially increasing the density of a polyurethane foam such as Reticel by thermomechanical compression should be avoided due to the destruction in mechanical properties.

### 4.4 Representative geometry

The above study, and those simulations referenced throughout this work, used full scale head forms with realistic (if simplified geometry), namely the curvature of the skull. This work has been based on the performance of the materials without analysis of shape or representative skull (including a rear face) and therefore is likely to exhibit different behaviour to systems of higher biofidelity, as blastwave interaction will be more complex as highlighted by Wilgeroth et al [12].

A jig forming a closer approximation to head geometry in addition to material properties would provide a much more accurate representation, as it would be able to capture the blastwaves reflecting off the inner contour of the skull. The jig used in TS2 was designed to simulate a transducer arbitrarily placed at the centre of the brain, but this not necessarily correlate to the most vulnerable area, such as the cerebellum [7].

Both jigs featured in this work were fixed relative to the support stanchion, where literature has shown that restrained ‘heads’ suffer less damage (particularly fewer deceleration injuries). More representative setups should include neck joints to allow the same range of movement as the human head. This would introduce acceleration-deceleration forces, which although more analogous to an explosive event would be more complicated to analyse.

### 4.5 Blast mitigating materials

The mass-volume constraints in helmet design restrict the doctrine of blast mitigation that can be applied as conventional techniques of thick cladding cannot be employed. Additionally, other functional requirements such as voids for ear protection and communication systems conflict with coverage. If foam cannot achieve the desired effect within these constraints, then a different method of design should be employed.

An example of a departure from traditional structure is the Multi-directional Impact Protection System (MIPS) helmet, a concept which is being employed across many areas of sports including cycling, snow sports, American football, and motorcycle helmets. The design utilises two layers with a low-friction material in between, to allow the inner layer to ‘float’ in the same way that the brain does within the CSF. Any impact on the outer layer resulting in movement cause the outer layer to move with respect to the inner layer, dissipating the energy in the sandwiched material, resulting in far less energy being coupled to the head [49].

Non-Newtonian or Shear Thickening Fluids (STF) have also seen a rise in popularity of use [50]. Recent developments in the field of snowsports equipment have seen the emergence of a beanie or wool hat impregnated with STF, which can be worn in place of a ski helmet. The final evolution of the design is expected to comply with ASTM F2040 and EN1077 safety standards and weigh less than a conventional ski helmet at 217 g. It is thought to be more comfortable as the weight is distributed more evenly around the head, and at 23 mm thick could be a strong candidate for implementation within a combat helmet, providing comfort as well as protection [51]. Whilst this example looks at blunt impact protection purposes only, there is early evidence to suggest that STFs also exhibit promising blast mitigation behaviour, but to the authors knowledge this simulation has not been experimentally validated or structured in a representative 3D manner [52].

Other novel materials to consider are Auxetic foams, a group of materials with negative Poisson's ratios. This feature increases the material stiffness under compression compared to the base foam, as the re-entrant cell walls prevent ‘bottoming out’, affording greater energy absorption capabilities [53, 54, 55].

## 5 Conclusions

The following general and specific conclusions have been drawn from this work:

1. **The denser the foam, the greater the amplification effect it generates.** Increase in density correlates with increase in normalised impulse as a result of increase in peak overpressure, observed across a range of samples of varying material in order to increase the density range of the population. Thickness and volume were not observed to have any effect within the range trialled here in TS1.
2. **Increase in mass rather than decrease in volume is the underlying influencer.** The results of TS2 show that an increase in density as a result of reduction in volume does not have an influence on the performance of the foam, therefore it can be reasonably concluded that the increase in mass is the cause. It can also be observed that comparable performance to a currently available comfort pad can be obtained with a foam of much lower density, showing potential for a significant mass saving effect.
3. **Polyurethane foams cannot fulfil the requirements within the mass-volume envelope for combat helmets.** Whilst foams are reported to achieve successful blast mitigation once they are employed at a certain thickness, this work has shown that many off the shelf polyurethane open cell foams cannot achieve adequate performance within the tight mass-volume envelope imposed by combat helmets, and that different material should be investigated. Additionally, a desired density or thickness should not be achieved by thermo-mechanically altering the foam post manufacture, as this results in complex gradiated structures with changes in relative density throughout, and a more brittle structure capable of absorbing less energy. It should be noted however that the range of material properties trialled during this work is very narrow, and that only one source of foam from a single manufacturer was used. in TS2.
4. **Simplification of the system changes the behaviour, making it difficult to extrapolate.** This study allowed for the study of blast in isolation only, and the fixed nature of the jigs prevented any movement of the head approximation. This ensured that the only mechanism of damage that could occur was that of primary blast injury caused by the blastwave itself. Whilst this allows for clearer understanding of the specific mechanism of transmission it is not representative of a real explosive event with fragmentation and gross body displacement. The complex geometry of the skull should also be considered in future work to capture the interactions of the rebounding shockwaves.
5. **Helmets that are not design with blast mitigation properties in mind may increase the risk of bTBI despite protecting against blunt and ballistic threats.** This is compounded by the lack of consensus as to which mechanism of damage carries the greatest risk of injury. If the underlying behaviour of shockwave transmission into the brain is not well understood, it is very difficult to design a system to defeat it, and to embed it into a helmet with other conflicting requirements. The MIPS concept would not be suitable for attaching the multiple sensors and vision aids that are becoming prevalent in modern dismounted warfare for example, and voids must be maintained around the ears to allow integration of communication equipment. Retrofitting a blast mitigation layer to a system that was fundamentally designed for impact and ballistic protection may not be possible, in which case the industry may need to design for purpose in the first instance. If mitigating against all types of insult cannot be achieved, then using different helmet variants for different roles (where possible) may be the most effective solution based on the most likely threat or greatest element of risk in the given situation.

## Declarations

### Author contribution statement

S. Bloodworth-Race: Conceived and designed the experiments; Performed the experiments; Analyzed and interpreted the data; Wrote the paper.

R. Critchley: Conceived and designed the experiments; Analyzed and interpreted the data; Contributed reagents, materials, analysis tools or data.

A. Peare: Performed the experiments; Contributed reagents, materials, analysis tools or data.

R. Hazael: Conceived and designed the experiments; Analyzed and interpreted the data; Contributed reagents, materials, analysis tools or data.

T. Temple: Contributed reagents, materials, analysis tools or data.

### Funding statement

This research did not receive any specific grant from funding agencies in the public, commercial, or not-for-profit sectors.

### Data availability statement

Data will be made available on request.

### Declaration of interests statement

The authors declare no conflict of interest.

### Additional information

No additional information is available for this paper.
